# Forecasting thermal stress for sports tourists at the 2026 FIFA World Cup

**DOI:** 10.1007/s00484-024-02785-4

**Published:** 2024-09-28

**Authors:** Christopher Craig, Ismail Karabas

**Affiliations:** Murray State University, Murray, KY USA

**Keywords:** Wet Bulb Globe Temperature, Climate change, Football, Soccer, Sports tourism

## Abstract

This study explores thermal stress conditions (i.e. heat, humidity, sunlight exposure) sports tourists can expect when attending the 2026 FIFA World Cup. Sports tourism’s growth is driven by international mega-events like FIFA World Cups and Olympic Games. With planning ongoing for the 2026 FIFA World Cup football (soccer) tournament, what remains unstudied are the heat conditions spectators can expect at the 16 host stadiums in the United States, Mexico, and Canada. The inquiry is important considering (1) the tournament is taking place in warm-weather months of June and July, (2) thermal stress contributes to heat-related illnesses like heat stroke, and (3) many destination-bound tourists will be at elevated risk to heat. Accordingly, we report historical and forecasted thermal stress levels documenting expected conditions at each host stadium. Notably, forecasts indicate worsening thermal stress compared to long-term means, providing support for the study’s methodology. Practical implications and limitations are provided.

## Introduction

The purpose of this study is to investigate the thermal stress conditions (i.e. heat, humidity, sun exposure) sports tourists can expect to encounter at the 2026 FIFA World Cup football (soccer) organized by the International Federation of Football Association (i.e. FIFA). Sports tourism is among the fastest growing subsectors of tourism internationally. For example, “major sporting events, such as the Olympic Games, football and rugby championships have become powerful tourism attractions themselves” (UNWTO 2023a: par. I). Sports tourism involves overnight travel away from one’s residence for the primary purpose of attending a sporting activity (Weed 2009). Sports tourism provides economic incentives to host-locations including hospitality (i.e., bars, hotels, restaurants), shopping, and other tourist attractions (Mulyanto 2023). One of the biggest sports tourism events in recent history was the 2022 FIFA World Cup in Qatar. According to the UNWTO (2023b), after hosting the tournament, international arrivals increased by 95% into 2023. With an additional 16 nations participating in the 2026 FIFA World Cup hosted by the United States, Mexico, and Canada (FIFA 2023), the global and local economic impacts are expected to be even greater than the 2022 tournament.

Sports tourism involves a variety of stakeholder groups including spectators, hosts/destination managers, and participants (Weed 2009). Here, our focal group is spectators attending football matches at the 16 stadiums for the 2026 FIFA World Cup. There are multiple motivations that spectators have for traveling to and attending a sporting event (Weed and Bull 2012). Among the most salient motivators are affinity for a sport, team, or athlete (King and Karabas 2024; Kurtzman and Zauhar 2005). Another factor influencing tourists—irrespective type of tourism—is climate change and its consequences (e.g. extreme heat) (Scott et al. 2019). Weather entails the short-term meteorological conditions experienced over the span of minutes to months (i.e. what tourists will experience onsite at an event). A recent review article reveals that warming temperatures from climate change are an area of concern in the sports tourism literature (Orr et al. 2022). Researchers have primarily focused on the athlete (e.g. health, performance), with far less attention given to spectator physical interaction with the natural environment (Dingle 2009; Orr et al. 2022).

Yet, when sports tourism occurs during warm-weather months, sports tourists spectating events are at heightened risks to thermal facets of the atmosphere, otherwise known as thermal stress (Nowak et al. 2022; Orr et al. 2022). Consequences of thermal stress include heat exhaustion, heat stroke, hyperthermia, and exertional heat illness (Orr et al. 2022). Thermal stress risks are further intensified by warming temperatures with a changing climate (Nowak et al. 2022; Orr et al. 2022). The observed effects of climate change (e.g. intensifying thermal stress) underscore a growing need for “responsible management practices for mega events” (Otto and Heath 2009: 169). With preparations in progress for the 2026 FIFA World Cup, what remains unstudied are the thermal stress conditions to be expected at each of the tournament’s 16 host stadium locations. Understanding of thermal stress can assist with preparation for destination-based tourists who (1) make travel decisions far in advance (Gary 2024), (2) are motivated by non-weather factors (e.g. Kurtzman and Zauhar 2005; Weed and Bull 2012), and (3) may be unacclimated to local thermal stress conditions (e.g. Matzarakis et al. 2018).

The remainder of this section describes literature related to tourism, thermal stress, and our focal sports tourist (i.e. spectators) followed by our operationalization of thermal stress with research questions. Next are material and methods, results and analysis, and discussion sections.

### Tourism, thermal stress, and sports tourists

This study is grounded in and builds upon thermal stress studies conducted by the tourism discipline, which have primarily focused on acceptable conditions for tourism performance (e.g., visits). For instance, Perkins and Debbage (2016) conducted a survey and inferred that tourists had a “potentially high aversion” to visitation at the Phoenix (United States) zoo during extreme thermal stress conditions. In an urban environment, Karimi and Mohammad (2022) found that urban centres with high thermal stress were viewed unfavarouble by the majority of tourists (> 60%) who were surveyed while visiting the centres. In fact, there have been a number of studies that have explored micro-climates of destinations measured as thermal stress such as Hungary, Luxemburg, Poland, and Spain (Karimi and Mohammad 2022; Matzarakis et al. 2013; Rozbicka and Rozbicki 2021; Vitt et al. 2015), using retrospective and/or future-oriented forecasts based on climate models for the distant future (e.g., 2071–2100). Like the broader tourism climatology literature (e.g. Rutty et al. 2020), thermal stress studies have primary focused on acceptable conditions for tourism, not conditions that could pose imminent health and safety risks from heat exposure. We expand upon the tourism literature’s study of thermal stress by focusing on (1) health and safety risks from thermal stress, and (2) shorter-term forecasts that more accurately describe conditions to be expected at a destination. We extend this study within the context of sports tourism.

Sports tourists spectating events are often at greater risks to thermal stress than athletes. For instance, heat was observed 15 °C higher outside the stadium during the IAAF World Championships than inside the stadium where competition occurred (Bermon and Adami 2019). Increased risks for spectators are largely attributable to (1) short acclimation periods and (2) lack of understanding about how to counteract heat (e.g., hydration schedules, proper attire) (Matzarakis et al. 2018). Risk seriousness for the 2026 FIFA World Cup is underpinned by the increased occurrence of heat-related tourist deaths in the United States attributable to extreme conditions (e.g. Zerkel 2023). In fact, “excessive heat is the leading weather related killer in the United States” among all facets of weather, including floods, hurricanes, and tornados (National Weather Service 2023: par I).

There are two recent mega-events where researchers explored historical thermal stress conditions for hosting geographies: the 2020 Olympic Games in Tokyo (Kakamu et al. 2017; Matzarakis et al. 2018) and the 2022 FIFA World Cup in Qatar (Matzarakis and Fröhlich 2015). Kakamu et al. (2017) compared 2016 thermal stress for Tokyo to the three prior Olympic Games locations in 2016, 2012, and 2008—Beejing, London, Rio De Janeiro, respectively—finding that heat conditions were comparatively inferior for sports tourists in Japan, including visitors. Matzarakis et al. (2018) also conducted retrospective analysis for Tokyo finding thermal stress conditions were heighted during the months in which games were eventually held, July and August. Matzarakis and Fröhlich (2015) analysed historical meteorological data for the 2022 FIFA World Cup in Qatar, observing extreme thermal stress risks from May to September. Findings were consistent with FIFA’s own feasibility studies that indicated the tournament was not viable during the proposed months of June and July due to extreme heat (Brennan 2022). Against precedent, FIFA moved the tournament to the winter months of November and December to reduce thermal stress risks. Methodologically, we build on prior visitor-oriented study’s as the first known to forecast thermal stress conditions spectators can expect at a mega-event (i.e. 2026 FIFA World Cup).

Ultimately, the technical requirements on the applications to host the 2026 FIFA World Cup bid did not require detailed weather information of applicant stadiums, inside or out (FIFA 2017). However, FIFA does conduct its own diligence which resulted in moving the start-date of the 2022 FIFA World Cup to November (Brennan 2022). For the upcoming North American iteration of the tournament, FIFA is considering “heat and humidity” for each of the host cities when determining match times when the schedule is drawn in 2025 (Bonagura and Carlisle 2024; Gary 2024). With dates and locations set, the most viable option will be holding matches during the daytime hours for stadiums in locals with lower “heat and humidity” conditions and/or at climate controlled (designated ^CC^ in Table 1) stadiums.

**Table 1:** Football match locations and corresponding date ranges

#	Location	Group Stage	Round of 32	Round of 16	Quarter-Finals	Semi-Finals	Bronze	Final
1	Atlanta^cc^	5	1	1		1		
2	Boston	5	1		1			
3	Dallas^cc^	5	2	1		1		
4	Houston^cc^	5	1	1				
5	Kansas City	4	1		1			
6	Los Angeles^cc^	5	2		1			
7	Miami	4	1		1		1	
8	New York	5	1	1				1
9	Philadelphia	5		1				
10	San Francisco	5	1					
11	Seattle	4	1	1				
12	Guadalajara	4						
13	Guadalupe	3	1					
14	Mexico City	3	1	1				
15	Toronto	5	1					
16	Vancouver^cc^	5	1	1				

^ŧ^*n* = 104 games; Group stage: June 11–27; Round of 32: June 28-July 3; Round of 16: July 4–7; Quarter-Finals: July 9–11; Semi-Finals: July 14–15; Bronze: July 18; Final: July 19; *cc* = climate-controlled

Shifting games later in the day promotes the health and safety of athletes and spectators once a match has begun, though later start times due to thermal stress have the potential to extend heat exposure to fans outside of stadiums. Furthermore, heat exposure could also be heightened at the five climate-controlled stadiums when hosting daytime matches, where tourists travel to and/or congregate outside of stadiums. Climate-controlled settings and stadium characteristics (e.g. covered seating) will reduce heat exposure once spectators are inside some stadiums. Yet, for spectators with seats directly exposed to sunlight, who walk long distances to stadiums, who participate in recreation outside stadiums (e.g. FIFA Fan Fest ™), or remain outside the stadium for other reasons (e.g. security lines), heat illness risks from thermal stress are heightened (e.g. Global Heat Health Network 2024).

### Operationalizing thermal stress

The study’s measure of thermal stress is the Wet Bulb Globe Temperature (WBGT) (National Weather Service 2023) equation (°C). Meteorological variables captured by the equation include cloud cover/solar radiation, humidity, temperature, sun angle, and wind speed (National Weather Service 2023). The WBGT found its origins in the 1950s in the United States military to control heat illness during training, and was updated in the 1960s using epidemiological analysis to identify dangerous WBGT levels (Budd 2008). Observational studies have since documented that elite athletes are not as impacted by thermal stress measured as WBGT compared to non-professional recreationalist (e.g. Montain et al. 2007). The WBGT has been used to assess weather risks for tourism in Australia (Skinner et al. 2001) and was recently applied to predict tourists’ arrivals to the island state of Hawaii in the United States (Craig and Oxarart 2024). The WBGT remains the most widely adapted measure of thermal stress risk (e.g. Budd 2008). For example, it is monitored in the United States for the purposes of worker and recreationalists (professional or amateur) safety to estimate when heat illness is likely (National Weather Service 2023).

The study’s operationalization is grounded in extreme value theory, which posits by nature extremes are anomalous (i.e. infrequent) and require advanced methods for detection and analysis (Embrechts et al. 2013). A key facet of the theory that makes it applicable to our study is that it considers probabilities of outcomes (e.g. health or safety) which are often challenging to calculate dependent on data resolution (Galambos 1994). The theory has been applied to weather extremes, including weather extremes of consequence to outdoor tourism activities (e.g. Marty and Blanchet 2012). Comparably, we adapt the theory’s peak over threshold method—or counts over a threshold (Embrechts et al. 2013)—to capture thermal stress risks (or thresholds) that can be expected at the 2026 FIFA World Cup for spectating sports tourists. The thresholds have previously been epidemiologically prescribed for intermittent recreationalists (Roberts et al. 2021), which includes sports tourists engaged in recreation while attending events. Compared to retrospective and forecasts models based on climate change scenarios in the distant future, our application of extreme value theory with short-term forecasts will allow us to more accurately determine risks probabilities sports tourists can expect in lieu of only reporting retrospective findings (e.g. Matzarakis et al. 2018).

The use of the WBGT is advantageous to our study for two reasons. First, WBGT levels are epidemiologically-derived (i.e. empirically grounded) for different types of work and recreation when activities need to be altered or discontinued to maintain safe conditions (e.g. National Weather Service 2023). And second, WBGT forecasts are widely available in the United States with site-specific forecasts available up to seven-days in advance (National Weather Service 2023). We acknowledge the WBGT has its limitations such as (1) clothing can counteract health and safety risks, (2) WBGT measurements are taken in direct sunlight, and (3) other indices have proven slightly more predictive of heat illness (Budd 2008; National Weather Service 2023; Thorsson et al.^2021^). Though, we defer to WBGT because of its prescriptive nature (e.g. levels at which recreation should be discontinued) and widespread accessibility of forecasts to the general public (National Weather Service 2023).

This study contributes to the sports tourism literature as the first known to forecast thermal stress (WBGT) conditions that sports tourists can expect when attending an event. With heat conditions worsening as a process of climate change—and with heat records routinely being broken in recent years (e.g. National Oceanic and Atmospheric Administration [NOAA] 2024a)—it is no longer sufficient to report historically conditions. Specifically, we will build forward facing WBGT forecasts based on daily historical thermal stress conditions (1984–2023) at each of the 16 host stadium locations for the 2026 FIFA World Cup. Study findings will answer our guiding research question:

*Research Question:* What thermal stress conditions can sports tourists expect when attending the 2026 FIFA World Cup (June and July 2026)

## Materials and methods

The WBGT equation consists of three variably weighted terms including wet bulb (70%) temperature, (20%) black globe temperature, and (10%) dry temperature (National Weather Service 2023). Unlike other variably weighted tourism index equations (e.g., Holiday Climate Index-Beach and -Urban, Rutty et al. 2020), WBGT assesses weather risks, not weather favourability. The first and last terms are retrievable using NASA’s (2024) data access viewer database. Black globe temperature is not commonly reported by meteorological stations, though the three variables needed to estimate the variable are dry temperature, relative humidity, and short-wave all sky solar radiation. See Table 2 for black globe and WBGT equations (Hajizadeh et al. 2017).

**Table 2:** Equations, variables, and units

*Equation 1.* T_B_ = 0.01498SR + 1.184T_D_ – 0.0789RH – 2.739
*Equation 2.* WBGT = (0.7*T_W_) + (0.2*T_B_) + (0.1*T_D_)
WBGT = wet bulb globe temperature (°C)
T_W_ = wet bulb temperature (°C)
T_B_ = black globe temperature (°C)
T_D_ = dry temperature (°C)
SR = solar radiation (watt/meter^2^)
RH = relative humidity (%)

**Fig. 1:**
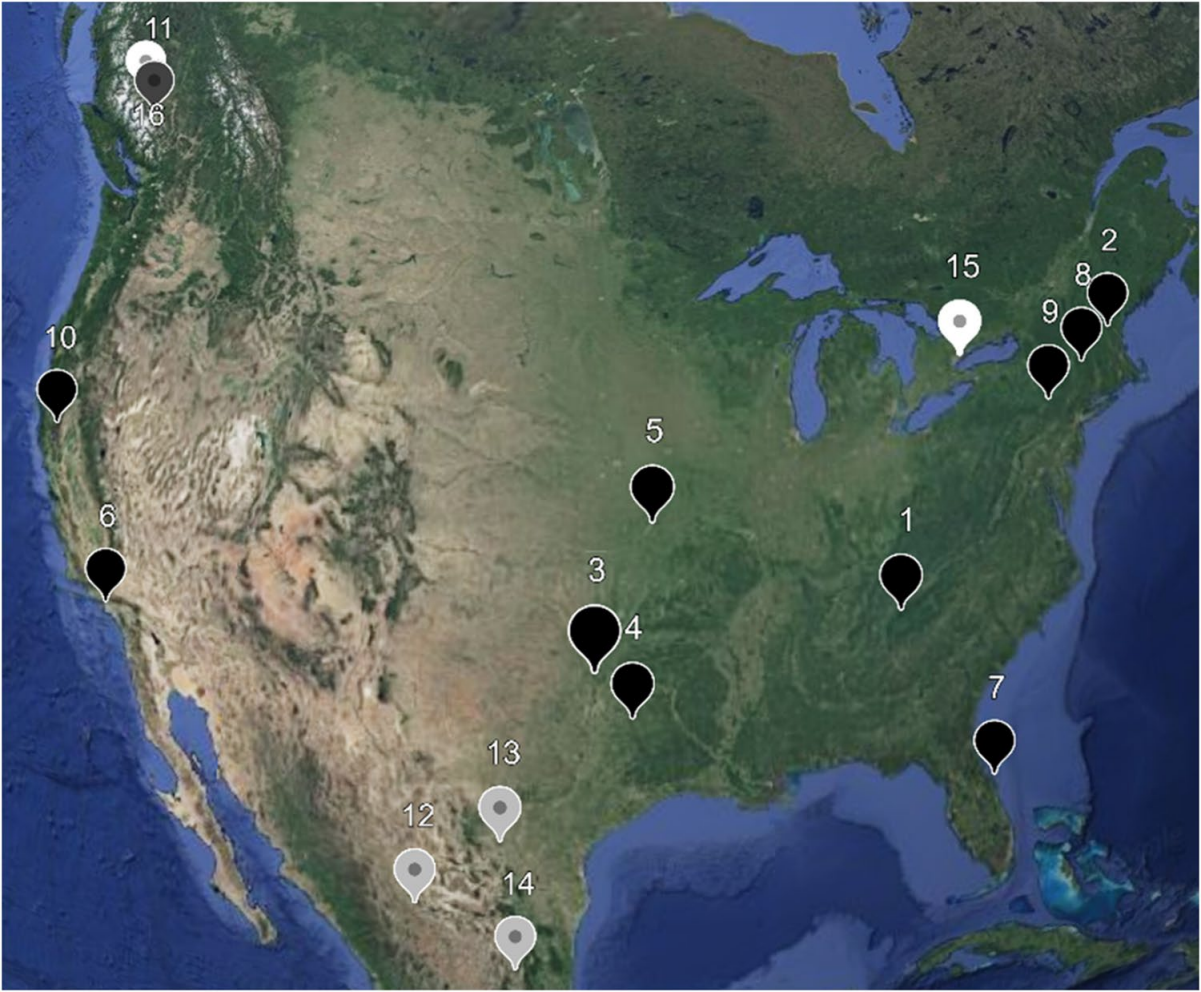
World Cup 2026 stadium locations interactive map. *Note. Black = *United States; Grey* = *Mexico; White* = *Canada; [Insert KML link upon acceptance]*

Daily data needed to calculate the equations was retrieved 1984 to 2023 (*n* = 40 years, *n* = 14,610 days per location) corresponding with 16 stadium coordinates (Fig. 1). WBGT risk levels are provided in Table 3 for non-acclimated and acclimated intermittent recreationalist from Roberts et al. (2021) as adapted by the National Weather Service (2023). To test our *Research Question*, we created future-oriented WBGT model forecasts for each of the 16 stadium locations using the expert modeler forecasting add-on to IBM SPSS v. 29 statistical software. Expert modeler selects a best-fit forecast model (i.e. simple, exponential smoothing, or ARIMA model) based on retrospective analysis of a timeseries. Models were forecasted through July 2026 to provide expected WBGT conditions at each host location while the tournament is ongoing. Model dependent variables include mean monthly WBGT (°C) and Level 0-Level 4 mean monthly risk days (count) from Table 3. The number of Level 5 observations (Dallas *n* = 7, Houston, *n* = 1) were not adequate to be detected by models.

**Table 3:** Risk levels and WBGT ranges for intermittent recreation dependent on acclimation

Risk	WBGT (°C)	Non-acclimated	Acclimated
Level 0	< 18.4	Minimal risk	Minimal risk
Level 1	18.4–22.3	Increased risk	Minimal risk
Level 2	22.3–25.7	Moderate risk	Increased risk
Level 3	25.7–27.9	Moderate to high risk	Increased to moderate risk
Level 4	27.9–30	Very high risk	Moderate to high risk
Level 5	> 30.0	Extremely high risk	Extremely high risk

## Results and analysis

We first calculated historical thermal stress levels for WBGT from 1984–2023 (*n* = 14,610 daily observations) for each of the 16 host stadium locations in Fig. 1. Table 4 provides historical means for WBGT (°C) and each of the risk levels (Level 0-Level 4). Next, we ran 83 retrospective timeseries models using stadium coordinates for the dependent variables monthly mean WBGT (°C) and mean monthly risk level days (count). Model statistics are provided in the APPENDIX Table 6. Using expert modeler in IBM SPSS v. 29, best-fit models emerged for each location. In order of frequency, there were four types of timeseries models: (1) simple seasonal, (2) Winters’ additive, (3) seasonal ARIMA, and (4) Winters’ multiplicative. The emergence of multiple models-types demonstrates the utility of not preselecting any one model when analysing weather timeseries at multiple locations.

**Table 4:** Stadium locations and mean monthly WBGT observations (June and July, 1984–2023)

		June Mean Observations (1984–2023)						July Mean Observations (1984–2023)					
#	Location	Mean	Level 0	Level 1	Level 2	Level 3	Level 4	Mean	Level 0	Level 1	Level 2	Level 3	Level 4
1	Atlanta^cc^	22.60	2	10	17	2	0	24.47	0	4	20	8	0
2	Boston	17.27	18	10	2	0	0	20.80	6	16	9	1	0
3	Dallas^cc^	25.17	0	2	14	11	2	26.84	0	0	5	18	7
4	Houston^cc^	26.22	0	0	9	18	3	26.93	0	0	3	24	5
5	Kansas City	21.73	5	11	12	2	0	23.89	1	7	14	7	1
6	Los Angeles^cc^	16.84	23	7	0	0	0	19.37	10	18	2	0	0
7	Miami	26.64	0	0	4	25	1	27.40	0	0	0	23	8
8	New York	18.35	14	12	4	0	0	21.33	4	14	11	1	0
9	Philadelphia	19.78	10	13	7	0	0	22.73	2	11	15	3	0
10	San Francisco	14.66	28	2	0	0	0	16.35	26	5	0	0	0
11	Seattle	13.82	28	2	0	0	0	16.55	24	6	1	0	0
12	Guadalajara	20.08	2	28	1	0	0	19.23	4	27	0	0	0
13	Guadalupe	23.87	0	4	24	3	0	23.57	0	4	26	1	0
14	Mexico City	13.44	30	0	0	0	0	12.97	31	0	0	0	0
15	Toronto	15.12	24	5	0	0	0	18.71	14	13	4	0	0
16	Vancouver^cc^	11.52	29	1	0	0	0	14.59	28	3	0	0	0

^ŧ^Due to rounding, there are some observations less than the number of days in a month

Simple seasonal models are appropriate for data with no trend but a constant seasonal effect over time (IBM 2022). Winters’ additive models are appropriate for data with a linear trend and a constant seasonal effect over time, but do not depend on past levels (IBM 2022). Unlike the others, ARIMA are not a type of exponentially smoothed model. ARIMA models have three terms, autoregressive (AR; level), differencing (I; season), and moving average (MA; trend) and include non-seasonal and seasonal components for each denoted as (p,d,q) (P,D,Q) (IBM 2022). For example, the Level 4 model for Miami indicates: a single order (i.e. one month) of autoregression to the non-seasonal component (p), a single order (i.e. 12-months) of the seasonal difference component (D) is required to stationize the data, and a single order (i.e. 12 months) to the moving average seasonal component (Q). For example, (p) July’s Level 4 risk days are influenced by June’s, (D) a 12-month differencing is required to remove seasonality from data, and (Q) 2023’s risk days are influenced by 2022’s. Winters’ multiplicative models are appropriate for data with a linear trend, a constant seasonal effect over time, and that depends on past levels (IBM 2022).

The stationary R^2^ (*St. R*^*2*^) statistic provides a comparative measure of fit for models, in that it compares model fit to a generic baseline model’s fit (IBM 2022). Each model that emerged outperformed the baseline model, with *St. R*^*2*^ values ranging from 0.13 to 0.81. In fact, 89% of the observed models had a *St. R*^*2*^ > 0.50 value. *R*^*2*^ values generally demonstrate good fit across models, with the exceptions for locations that had limited number of observations for more extreme thermal stress risk levels (e.g. Level 3 and Level 4). For instance, the Guadalupe model for Level 3 risks had an *R*^*2*^ value of 0.03 derived from very few mean monthly observations (*n* = 3 in June and *n* = 1 in July) (Table 4). Given widespread acceptable model fit, we forecasted dependent variable values from January 2024 to July 2026 to capture expected values for June and July 2026.

The resultant forecasted values document what monthly WBGT (°C) and risk level days (count) can be expected at the 16 participating host stadiums (see Table 5). There are two irregularities from Tables 4 and 5 that warrant explanation. First, values in Table 4 are rounded to whole days, which led to some locations reporting a lower number of observations than days in the month (*n* = 30 for June, *n* = 31 for July). A comparable phenomenon occurred in Table 5, where there were months with more and less days than days in the month due to forecasting ranges. And second, Table 5 has negative forecasted values, which is not possible but does provide an indication of the high unlikelihood of occurrence. There are several key takeaways that emerged from forecasts, including comparisons of forecasts (Table 5) to observations (Table 4):

1. For mean WBGT (°C), all values were higher for forecasts than observations (between 0.35° to 2.18 °C).
2. Two of the four hottest host locations (i.e. Houston and Dallas) have climate-controlled stadiums, and the other two have extended shade over stands (i.e. Miami and Guadalupe).
3. Six host locations have mean forecasts (°C) of Level 0 for June 2026 and four locations for July 2026.
4. Seven host locations are forecasted to have more thermal stress risk days (varying levels) in June 2026 compared to observations, and 11 are forecasted to have more thermal stress risk days (varying levels) in July 2026 compared to observations. For June, most stadiums (57%) are climate-controlled, though in July—when the finals schedule begins—a majority of stadiums are not (82%).
5. The three most profound differences between forecasts and observations in thermal stress risk days (varying levels) are in Miami, Houston, and New York. The biggest difference is in Miami, where 12 more Level 4 risk days (60%) are forecasted (*n* = 20) than the observational mean (*n* = 8). Restated, the likelihood of attending a football match on a Level 4 risk day is 60% more likely than history would suggest.

**Table 5:** Stadium locations and mean monthly WBGT forecasts (June and July, 2026)

		June Forecasts (2026)						July Forecasts (2026)					
#	Location	Mean	Level 0	Level 1	Level 2	Level 3	Level 4	Mean	Level 0	Level 1	Level 2	Level 3	Level 4
1	Atlanta^cc^	23.06	1	10	16	2	0	25.01	-1	3	19	8	0
2	Boston	18.42	17	10	2	0	0	22.05	3	16	13	1	0
3	Dallas^cc^	26.20	-1	2	14	12	3	27.96	-1	0	5	17	10
4	Houston^cc^	27.21	-1	0	8	17	7	27.98	-1	-1	2	19	12
5	Kansas City	22.71	4	12	12	2	0	24.97	0	8	14	8	0
6	Los Angeles^cc^	17.35	21	8	1	0	0	19.98	8	20	3	0	0
7	Miami	27.62	-1	-2	2	24	5	28.42	-1	-2	0	12	20
8	New York	19.44	13	12	4	0	0	22.49	2	14	16	1	0
9	Philadelphia	20.83	9	12	8	0	0	23.84	1	11	15	5	0
10	San Francisco	15.01	17	3	0	0	0	16.76	25	5	0	0	0
11	Seattle	14.50	27	2	0	0	0	17.31	23	7	1	0	0
12	Guadalajara	21.11	0	27	3	0	0	20.27	0	31	0	0	0
13	Guadalupe	24.86	-1	1	24	3	0	24.56	1	1	27	2	0
14	Mexico City	14.47	30	0	0	0	0	13.99	31	0	0	0	0
15	Toronto	17.21	22	7	0	0	0	20.89	3	20	7	0	0
16	Vancouver^cc^	12.43	29	1	0	0	0	15.59	27	4	0	0	0

^ŧ^Due to forecasts ranges, there are some observations less or greater than the number of days in a month

## Discussion

Study findings indicate that thermal stress is (1) intensifying irrespective 2026 FIFA World Cup host stadium location and (2) forecasted to be more intense than the long-term average (1984–2023). Risks vary from venue-to-venue, with some stadiums providing an environment where thermal stress concerns are at a minimum. For example, San Francisco, Seattle, Mexico City, and Vancouver (Table 5) all have mean WBGT (°C) forecasts and observations within Level 0 (nominal, < 18.4 °C) for both June and July. However, none of these locations are slated for hosting a match after the Round of 16. For many other locations—including all host stadium locations for the finals schedule—thermal stress is creating elevated risks (varying levels) for sports tourists travelling to attend events. Most notably is Miami where 60% more Level 4 risk days are forecasted than were observed (1984–2023). Miami is noteworthy because it will host a quarter-finals match as well as the bronze medal match in July. Another example is the finals host stadium location (i.e. New York) where Level 2 thermal stress risks are over 30% more likely than the historical mean.

Our study represents a methodological advancement over prior studies that have relied solely on retrospective analyses (e.g. Kakamu et al. 2017; Matzarakis and Fröhlich 2015; Matzarakis et al. 2018). For the 2022 World Cup in Qatar, prescriptions offered by Matzarakis and Fröhlich (2015) to move the tournament to November or later to avoid extremely dangerous thermal stress risks were adopted, though the influence of the study is unknown since FIFA conducted its own feasibility studies (Brennan 2022). Kakamu et al. (2017) and Matzarakis et al. (2018) both offered prescriptions to move event times for the 2020 Olympic Games in Tokyo that were not adopted, however. In these instances, forecasts would have provided tourists with more realistic expectations of onsite thermal stress conditions that could inform pre-travel planning. Grounded in extreme value theory (Embrechts et al. 2013), our shorter-term forecast methodology offers an advancement to prior operationalizations because risks probabilities (Table 5) (Galambos 1994) provide a more accurate depiction than retrospective or future-oriented forecasts with long temporal windows (e.g. 2071–2100). With the trajectory of climate change contributing to exponential heating conditions (e.g. NOAA 2024a), it is crucial that researchers provide the most accurate expectations possible for thermal stress once tourists are onsite at an event. Below, we will provide practical implications followed by limitations and future research directions.

### Practical implications

Study findings point to both supply- and demand-side implications. On the supply-side, the results pinpoint the host stadium locations where spectators can expect to experience the least risky heat conditions. These include venues where the host countries will be playing their first-round matches: Los Angeles (June 2026 WBGT forecast = 17.35 °C), Mexico City (June 2026 WBGT forecast = 14.47 °C), and Toronto (June 2026 WBGT forecast = 17.21 °C). Other locations with forecasted Level 0 risks are Seattle, San Francisco, and Vancouver. For tourists whose primary motivation is affinity of sport and not any particular team or player participating in the 2026 FIFA World Cup, these locations are where they can expect the safest heat conditions while attending a match.

There are far more matches where spectators are likely to experience some level of thermal stress risks, however. For instance, 67% of matches will be played in host locations with average WBGT forecasts over 18.4 °C, the threshold where Level 1 thermal stress risks begin (Table 3). Also, the entirety of the finals schedule will be played at host locations with elevated thermal stress risks (Table 1), including two matches in Miami where Level 4 risks are likely (Table 5). In higher risks scenarios, sports tourists should make plans prior to attending events to mitigate risks through measures such as procuring and packing protective clothing (Matzarakis et al. 2018). Tourists can also plan risk mitigative actions once onsite including tracking WBGT (National Weather Service 2023; NOAA 2024b) and setting hydration schedules (Matzarakis et al. 2018). If possible, spectators could purchase match tickets that (1) are not directly exposure to the sun and/or (2) have access to air-conditioned indoor spaces such as restaurants or bars.

There are also demand-side implications. First, for lower risk host cities (e.g. Los Angeles, Mexico City, Toronto), comparatively favourable weather conditions can be communicated to attract prospective sports tourists. Second, destination managers can use proven communication strategies to (1) attract tourists and (2) promote heat risk mitigation once tourists are onsite. Research has shown that general or generic messages are advantageous to concrete messages with longer booking windows (Kim et al. 2016). Considering the schedule will be drawn over a year in advance of matches (Gary 2024), destination managers of host locations with favourable thermal stress should begin by crafting and communicating abstract messages about weather to attract tourists. For instance, message content such as “Los Angeles has the best weather for football” would be superior to a more specific message. Destination managers can then employ a more concrete communication strategy closer to the event. This is particularly applicable for destination managers of host locations with higher thermal stress risk levels. An example concrete message could be “thermal stress risks are very high for next week’s football match in Miami (over 28 °C) so pack your sunscreen, a hat, and a refillable water bottle.” And third, destination managers can track WBGT (e.g. National Weather Service) and promote heat illness mitigation once tourists arrive at a match. Two mitigative measures are hydration and cooling stations outside and inside stadiums, particularly those not climate controlled. To monetize risk mitigation, host stadiums could sell heat protective clothing and products like reusable water bottles.

### Limitations

The primary limitation is that since the event is yet to occur, it is not possible to correlate thermal stress risks with (1) tourists’ destination decisions or (2) documented health illnesses or deaths that occur. Given the extraordinarily high demand for tickets, the months-long window to pre-purchase tickets, and historically high attendance at events, gameday weather will have nominal impact on attendance (i.e. recreationalist and tourist flows). Once matches occur, researchers should assess the number of heat related illnesses and deaths. A second limitation is that forecasts and historical observations for thermal stress are reported monthly. Yet, on the day of matches, weather is indeterminate. An interesting line of future inquiry would be to explore ways to engage tourists with forecasts prior to arriving at a destination (e.g. two-week WBGT forecasts provided by the National Weather Service (2023)).

Third, the WBGT has several documented deficiencies (e.g. Budd 2008; Thorsson et al. 2021). The deficiencies are related in part to overestimation and lower correlations between WBGT and performance/health consequences. However, given the WBGT is the most widely reported and forecasted measure of thermal stress in the United States (National Weather Service 2023)—where the bulk of 2026 FIFA World Cup matches will occur—we believe it is judicious to err on the side of overestimating risks. Also, NOAA (2024b) recently launched an experimental interactive online tool called the “NWS Heat Risk” that provides seven-day forecasts for thermal stress risks based on the WBGT and its risks colour schemes. Fourth, there were some observations of poor model fit. Even in these instances, however, model fit compared to generic baseline models were acceptable (e.g. *R*^*2*^ = 0.02 and *St. R*^*2*^ = 0.51 in Guadalupe for Level 4). Future researchers should explore other methods that can improve model fit where limited number of observations exist. And lastly, the study considered thermal stress risk for intermittent recreation for tourists who are either non-acclimated or acclimated to local conditions. For spectators who do not have to engage in some type of recreation (e.g. walking to the stadium) and/or are sedentary once in a stadium, the risks of heat illness are lower than the WBGT would suggest (Japanese Society of Biometeorology 2023).

## Data Availability

Data is available for download from publicly accessible sources cited in the article.

## Appendix

Please see Table 6.

**Table 6:** Retrospective model statistics (1984–2023)

								Ljung-Box Q(18)			
#	Location	DV	Model type	*St. R* ^*2*^	*R* ^*2*^	*RMSE*	*MAPE*	*Statistic*	*df*	*p*	*Outliers*
1	Atlanta^cc^	WBGT	Simple Seasonal	0.72	0.95	1.70	214.65	15.78	16	0.468	0
		Level 0	Winters' Additive	0.74	0.96	2.56	21.52	23.03	15	0.084	0
		Level 1	Simple Seasonal	0.74	0.71	3.13	59.17	16.84	16	0.396	0
		Level 2	Simple Seasonal	0.76	0.86	3.31	44.21	20.99	16	0.179	0
		Level 3	Simple Seasonal	0.65	0.46	2.64	89.96	46.84	16	0.000	0
		Level 4	Simple Seasonal	0.78	0.05	0.25	87.73	8.54	16	0.931	0
2	Boston	WBGT	Simple Seasonal	0.71	0.97	1.56	66.39	17.40	16	0.360	0
		Level 0	Winters' Additive	0.65	0.94	2.33	20.99	32.03	15	0.006	0
		Level 1	Simple Seasonal	0.68	0.88	2.27	35.72	24.94	16	0.071	0
		Level 2	Simple Seasonal	0.69	0.67	2.13	64.97	8.24	16	0.941	0
		Level 3	Winters’ Additive	0.77	0.20	0.45	65.18	13.51	15	0.563	0
3	Dallas^cc^	WBGT	Simple Seasonal	0.71	0.96	1.53	24.49	18.01	16	0.323	0
		Level 0	Winters’ Additive	0.76	0.97	2.37	20.01	15.57	15	0.411	0
		Level 1	Simple Seasonal	0.71	0.67	2.68	58.21	17.79	16	0.336	0
		Level 2	Winters’ Additive	0.71	0.71	3.30	63.79	21.05	15	0.135	0
		Level 3	Simple Seasonal	0.73	0.76	3.40	78.80	8.28	16	0.940	0
		Level 4	ARIMA(0,0,1)(0,1,1)	0.47	0.52	3.24	93.97	6.67	16	0.979	0
4	Houston^cc^	WBGT	Simple Seasonal	0.71	0.95	1.44	7.42	15.28	16	0.504	0
		Level 0	Simple Seasonal	0.72	0.92	3.10	28.20	17.62	16	0.347	0
		Level 1	Simple Seasonal	0.71	0.61	3.13	67.61	20.59	16	0.195	0
		Level 2	Simple Seasonal	0.72	0.73	3.36	64.91	21.66	16	0.154	0
		Level 3	Simple Seasonal	0.66	0.84	3.86	50.95	23.28	16	0.106	0
		Level 4	ARIMA(0,0,1)(1,1,1)	0.42	0.51	2.95	85.73	24.75	15	0.053	0
5	Kansas City	WBGT	Simple Seasonal	0.72	0.96	1.92	64.88	21.98	16	0.144	0
		Level 0	Simple Seasonal	0.74	0.95	2.69	22.48	19.72	16	0.233	0
		Level 1	Simple Seasonal	0.76	0.71	2.72	56.67	27.82	16	0.033	0
		Level 2	Simple Seasonal	0.71	0.81	2.65	55.44	16.65	16	0.409	0
		Level 3	Simple Seasonal	0.66	0.63	1.84	74.10	24.74	16	0.075	0
		Level 4	ARIMA(0,0,1)(1,0,1)	0.27	0.27	0.86	79.33	15.03	15	0.449	0
6	Los Angeles^cc^	WBGT	Simple Seasonal	0.67	0.93	1.16	7.91	30.10	16	0.018	0
		Level 0	Simple Seasonal	0.67	0.83	3.75	22.96	39.74	16	0.000	0
		Level 1	Simple Seasonal	0.69	0.81	3.46	56.80	29.99	16	0.018	0
		Level 2	Simple Seasonal	0.73	0.33	1.75	74.27	13.93	16	0.604	0
		Level 3	Simple Seasonal	0.75	0.03	0.16	95.13	28.76	16	0.026	0
7	Miami	WBGT	Simple Seasonal	0.67	0.91	0.90	2.86	41.27	16	0.000	0
		Level 0	Winters’ Additive	0.72	0.56	2.47	78.91	26.30	15	0.035	0
		Level 1	Simple Seasonal	0.73	0.75	3.48	57.10	32.72	16	0.008	0
		Level 2	Simple Seasonal	0.69	0.68	4.98	62.75	16.85	16	0.396	0
		Level 3	ARIMA (0,0,1)(0,1,1)	0.39	0.84	4.56	45.93	8.75	16	0.923	0
		Level 4	ARIMA (1,0,0)(0,1,1)	0.50	0.72	3.12	104.87	27.38	16	0.037	0
8	New York	WBGT	Simple Seasonal	0.72	0.97	1.71	68.15	14.46	16	0.565	0
		Level 0	Winters’ Additive	0.71	0.94	2.41	19.70	15.87	15	0.391	0
		Level 1	Winters’ Additive	0.72	0.86	2.43	42.38	9.15	15	0.870	0
		Level 2	Simple Seasonal	0.65	0.73	2.26	64.11	9.22	16	0.904	0
		Level 3	Simple Seasonal	0.72	0.27	0.53	52.85	7.61	16	0.960	0
9	Philadelphia	WBGT	Simple Seasonal	0.72	0.97	1.70	151.63	11.79	16	0.758	0
		Level 0	Winters’ Additive	0.74	0.96	2.30	15.57	29.87	15	0.012	0
		Level 1	Simple Seasonal	0.73	0.79	2.69	50.10	11.64	16	0.768	0
		Level 2	Simple Seasonal	0.75	0.85	2.14	49.19	17.85	16	0.333	0
		Level 3	(0,0,1)(0,1,1)	0.50	0.50	1.09	65.57	11.88	16	0.752	0
		Level 4	(0,0,0)(1,0,0)	0.13	0.13	0.19	85.54	0.73	17	1	0
10	San Francisco	WBGT	Simple Seasonal	0.67	0.92	1.09	9.06	26.70	16	0.045	0
		Level 0	Simple Seasonal	0.68	0.47	1.99	4.81	22.45	16	0.129	0
		Level 1	Simple Seasonal	0.69	0.48	1.89	76.67	22.74	16	0.121	0
		Level 2	Simple Seasonal	0.75	0.05	0.32	90.04	21.55	16	0.158	0
		Level 3	Simple Seasonal	0.76	0.02	0.09	97.65	0.34	16	1	0
11	Seattle	WBGT	Simple Seasonal	0.70	0.94	1.24	37.39	20.60	16	0.194	0
		Level 0	Simple Seasonal	0.73	0.64	1.88	3.92	22.41	16	0.131	0
		Level 1	Simple Seasonal	0.75	0.66	1.79	67.96	21.63	16	0.156	0
		Level 2	Simple Seasonal	0.81	0.13	0.40	74.00	30.44	16	0.016	0
		Level 3	Simple Seasonal	0.76	0.02	0.05	97.67	0.36	16	1	0
12	Guadalajara	WBGT	Simple Seasonal	0.67	0.92	0.82	4.25	36.92	16	0.002	0
		Level 0	Simple Seasonal	0.59	0.92	3.38	36.25	25.04	16	0.069	0
		Level 1	Simple Seasonal	0.58	0.92	3.38	32.48	27.11	16	0.040	0
		Level 2	ARIMA(0,0,11)(1,1,1)	0.50	0.29	0.50	73.95	4.71	15	0.994	0
13	Guadalupe	WBGT	Simple Seasonal	0.68	0.95	1.14	5.90	18.41	16	0.300	0
		Level 0	Simple Seasonal	0.67	0.95	2.93	27.55	39.44	16	0.000	0
		Level 1	Simple Seasonal	0.67	0.67	3.88	69.16	27.42	16	0.037	0
		Level 2	Winters’ Additive	0.69	0.92	3.22	39.74	22.11	15	0.105	0
		Level 3	ARIMA(0,0,2)(1,0,1)	0.25	0.25	1.65	72.12	7.30	14	0.922	0
		Level 4	Simple Seasonal	0.51	0.02	0.05	97.67	0.00	16	1	0
14	Mexico City	WBGT	Simple Seasonal	0.68	0.89	0.79	6.47	44.00	16	0.000	0
		Level 0	Winters’ Multiplicative	0.81	0.98	0.13	0.13	42.66	15	0.000	0
15	Toronto	WBGT	Simple Seasonal	0.63	0.97	1.60	53.76	62.75	16	0.000	0
		Level 0	ARIMA(1,0,0)(0,1,1)	0.40	0.84	3.15	19.33	19.93	16	0.223	0
		Level 1	ARIMA(1,0,0)(0,1,1)	0.37	0.78	2.91	58.92	19.00	16	0.269	0
		Level 2	ARIMA(1,0,0)(0,1,1)	0.40	0.45	1.99	70.05	14.49	16	0.562	0
		Level 3	Simple Seasonal	0.70	0.05	0.22	89.51	12.76	16	0.690	0
16	Toronto	WBGT	Simple Seasonal	0.68	0.95	1.52	842.07	33.31	16	0.007	0
		Level 0	Simple Seasonal	0.72	0.46	1.39	2.29	17.44	16	0.357	0
		Level 1	Simple Seasonal	0.71	0.45	1.32	57.60	14.37	16	0.571	0
		Level 2	Simple Seasonal	0.79	0.05	0.23	94.04	26.73	16	0.045	0
		Level 3	Simple Seasonal	0.76	0.02	0.05	97.67	0.36	16	1	0
